# Population pharmacokinetics of intravenous and oral panobinostat in patients with hematologic and solid tumors

**DOI:** 10.1007/s00228-015-1846-7

**Published:** 2015-05-05

**Authors:** Marina Savelieva, Margaret M. Woo, Horst Schran, Song Mu, Jerry Nedelman, Renaud Capdeville

**Affiliations:** Novartis Pharma AG, CH-4002 Basel, Switzerland; Novartis Pharmaceuticals Corporation, East Hanover, NJ USA

**Keywords:** Panobinostat, Pan-deacetylase inhibitor, Pan-DACi, Population pharmacokinetics, Modeling, Bioavailability

## Abstract

**Purpose:**

The study aimed to characterize the population pharmacokinetics of panobinostat, a pan-deacetylase inhibitor that has demonstrated efficacy in combination with bortezomib and dexamethasone in patients with multiple myeloma.

**Methods:**

A nonlinear mixed-effect model was used to fit plasma panobinostat concentration-time data collected from patients across 14 phase 1 and phase 2 trials following either oral or intravenous (IV) administration. The model was used to estimate bioavailabilities of the two oral formulations and the effects of demographic and clinical covariates on the central volume of distribution and clearance of panobinostat.

**Results:**

A total of 7834 samples from 581 patients were analyzed. Panobinostat pharmacokinetic parameters were best characterized by a three-compartment model with first-order absorption and elimination. Bioavailability was 21.4 %. Median clearance was 33.1 L/h. Interindividual variability in clearance was 74 %. For Caucasian patients of median age 61 years, area under the curve (AUC) decreased from 104 to 88 ng· h/mL as body surface area (BSA) increased from the first to third quartiles, 1.8 to 2.1 m^2^. For Caucasian patients of median BSA 1.9 m^2^, AUC decreased from 102 to 95 ng· h/mL as age increased from the first to third quartiles, 51 to 70 years. For patients of median BSA and median age, AUC ranged across the four race categories from 80 to 116 ng· h/mL. Covariate analysis showed no impact on panobinostat clearance and volume by patients’ sex, tumor type, kidney function, liver markers, or coadministered medications. However, separate analyses of dedicated studies have demonstrated effects of liver impairment and CYP3A4 inhibition.

**Conclusions:**

Although covariate analyses revealed significant effects of body size, age, and race on panobinostat pharmacokinetics, these effects were minor compared to the interindividual variability and therefore not clinically relevant when dosing panobinostat in populations similar to those studied.

**Electronic supplementary material:**

The online version of this article (doi:10.1007/s00228-015-1846-7) contains supplementary material, which is available to authorized users.

## Introduction

Panobinostat is a potent pan-deacetylase inhibitor (pan-DACi) that increases the acetylation of proteins involved in multiple oncogenic pathways, including epigenetic aberrations, DNA replication and repair, cell cycle progression, protein metabolism and turnover, and tumor-cell survival [1]. It has shown promising antitumor activity in several tumor types [1, 2]. Fourteen open-label phase 1 and phase 2 studies of patients have evaluated the intravenous (IV) and oral use of single-agent panobinostat with multiple dosing schedules in patients with various solid and hematologic tumors [3–16]. Based on the phase 3 PANORAMA 1 trial, panobinostat has been approved by the US Federal Drug Administation (FDA) in combination with bortezomib and dexamethasone for the treatment of patients with multiple myeloma who have received at least two prior regimens, including bortezomib and an immunomodulatory agent.

An exploratory IV formulation and two oral formulations of panobinostat were developed, and the pharmacokinetics (PK) of each formulation has been extensively evaluated in patients during the past 10 years. Results from a phase 1 study of IV panobinostat in patients with refractory hematologic tumors demonstrated an approximate proportional increase in plasma concentration and drug exposure, with an estimated maximum concentration (*C*_max_, mean ± SD) of 565.6 ± 450.9 ng/mL at the highest dose administered (14 mg/m^2^) [6]. Oral panobinostat demonstrated rapid absorption (time to *C*_max_ <2 h), with a mean *C*_max_ of 71 ng/mL at 60 mg [17]. Early experiences demonstrated that oral panobinostat had a more favorable safety profile than the IV formulation. This, combined with the added dosing convenience, has led to oral formulation development and is the focus of the current analysis.

Two oral formulations were developed. The initial pilot clinical service formulation (CSF) was developed as a monohydrate salt with dry blend formulation and was used in two early phase 1 studies. This pilot formulation was subsequently modified to improve the manufacturing process by using a wet granulation of an anhydrous salt, which created the final market image (FMI) formulation for mass production and future commercialization. The in vitro dissolution profiles for the two oral formulations are similar at pH 2.0 and 4.5. Subsequently, ten studies, including six phase 2 studies, used the FMI formulation.

Panobinostat is metabolized through multiple pathways—including oxidation, reduction, hydrolysis, mono-oxygenation, and glucuronidation—into approximately 40 circulating metabolites [18]. These metabolites are devoid of inhibitory activity toward human pan-deacetylases. The oxidative metabolism of panobinostat is mediated primarily by CYP3A4 (70–98 %), with minor involvement of CYP2D6 and CYP2C19 [14]. Panobinostat shows a plasma protein binding of 90 %, independent of the drug concentration in plasma. Excretion of panobinostat and its metabolites takes place to a similar extent through the kidneys and liver, with 44.4 to 77.4 % of the dose recovered in the feces and 28.6 to 51.2 % of the dose recovered in the urine [18]. The fraction excreted unchanged in the urine was <2.5 % in patients with solid tumors with normal renal function [19].

Although its safety, efficacy, PK, and metabolism have been investigated in clinical trials as well as in several in vitro studies, a thorough analysis of panobinostat population PK has not been conducted. Population PK analyses are crucial in drug development to identify intrinsic and extrinsic factors among target populations that may contribute to variability in drug PK. Such variations may affect drug safety and efficacy. A population PK model is also an important tool for predicting exposure under new dosing regimens and as input to PK/pharmacodynamic (PD) models to predict clinical response. This study sought to evaluate the population PK of panobinostat using a data set from 581 patients across 14 phase 1 and phase 2 trials of patients with solid and hematologic tumors.

## Materials and methods

### Patient population

A population PK data set was constructed from 581 patients with advanced hematologic and solid tumors who participated in 14 phase 1 and phase 2 studies of panobinostat (Table 1). Studies A2101 and A2102 used IV panobinostat, whereas all other studies examined oral formulations of panobinostat. The CSF was used in two of the oral panobinostat studies, B2101and B2102, whereas the remaining studies used the FMI formulation. The two phase 1 dose-escalation studies of IV panobinostat used doses of 1.2 to 20 mg/m^2^ daily administered under various intermittent regimens. In three phase 1 dose-escalation studies, patients received oral panobinostat at doses ranging from 10 to 80 mg/day (administered weekly on days 1, 3, and 5; or every other week; or on days 1 and 4 every week). In five phase 2 studies, patients with cutaneous T cell lymphoma, chronic myeloid leukemia, and multiple myeloma received 20 mg panobinostat orally per day on days 1, 3, and 5 weekly. In three clinical pharmacology studies, patients received oral panobinostat at 20 mg under various regimens for study-specific purposes: in cycle 1 followed by 20 mg per day (days 1, 3, and 5 weekly) or 45 mg (days 1 and 4 weekly) in subsequent cycles. In a phase 2 study of Hodgkin lymphoma, patients received 40 mg panobinostat on days 1, 3, and 5 weekly.

**Table 1:** Summary statistics of baseline categorical covariates

Covariate	Distribution
Race	Caucasian, 496; black, 34; Asian, 27; other, 24
Sex	Male, 362; female, 219
Liver status	Normal, 483; mild, 91; moderate, 6; severe, 1

### Pharmacokinetic sampling and bioanalytical methods

PK sampling generally occurred from predose through 48 h after each oral dose or predose through 24 h after each IV dose. Panobinostat was precipitated from plasma samples, evaporated to dryness, reconstituted with 10 % aqueous acetonitrile (containing 0.2 % formic acid), and analyzed by liquid chromatography–tandem mass spectrometry assay. The assay was linear from 0.5 to 500 ng/mL [15], and the bias and coefficient of variation (CV) values of the quality-control sample results ranged from −0.7 to 0.7 % and 2.3 to 11.6 %, respectively. The lower limit of quantification of panobinostat was 0.5 ng/mL for all studies except B2201 and B2203, for which it was 0.1 ng/mL. Values below the lower limit of quantification (6 % of the total) were excluded.

### Construction of the population pharmacokinetics data set

The data sets for modeling were prepared using SAS version 9.2. The time of panobinostat dosing or collection of blood was computed based on the date and time when available. When the date or time was unknown, collection times were estimated based on time of examinations or schedule in the protocol. Demographic information, including age, body weight, height, sex, and race, was collected at screening and used in the analysis. For patients with missing baseline demographics (height [*n* = 35], body mass index [*n* = 35], creatinine clearance [CrCL] [*n* = 13], and body weight [*n* = 13]), median values of the population were used. BSA (m^2^) was computed via the Gehan–George formula as

$$ 234.94\times \left({\mathrm{Weight}}^{0.515}\times {\mathrm{Height}}^{0.422}\right)\ /10000 $$

where weight is in kilograms and height is in centimeters. CrCL (L/h) was computed via the Cockroft–Gault formula as

$$ \left(140-\mathrm{Age}\right)\times \mathrm{Weight}\times \left[0.85\ \mathrm{f}\mathrm{o}\mathrm{r}\ \mathrm{f}\mathrm{emales}\right]/72\times \mathrm{Creatinine}/88.4 $$

where creatinine was in micromoles per liter.

The data set was validated via double programming by an independent programmer.

### Data analyses and development of the population pharmacokinetic model

The model was developed based on the methods described by Wahlby and colleagues [20]. Scatterplots used to visualize the plasma concentration vs. time profile were generated using R version 2.8.1 and SAS version 9.2. PK model building was conducted using the first-order conditional estimation method with INTERACTION (FOCEI) option, using the default three significant digits for convergence, within the software program nonlinear mixed-effect modeling (NONMEM) extended version VI and version VII. Random interindividual variability parameters were estimated using exponential parameterization. The exponential error models ensured that parameter estimates were within reasonable physiological bounds; negative values were not possible. Model selection criteria were based on both objective function values (OFVs), as generated by NONMEM, and graphic diagnostic plots. For each model analyzed, NONMEM calculated the minimum value of the OFV—a statistic that, up to an additive constant, is minus twice the log likelihood of the data. When two models are nested and the additional parameters of the larger model are not needed, the difference in their minimum OFV is asymptotically *χ*^2^ distributed, with the number of degrees of freedom equal to the number of parameters not common to the two models. The likelihood ratio test compared the two models on the basis of the difference in their OFV values.

Four main diagnostic scatterplots were used in the model selection processes: population-predicted vs. observed concentrations, population-weighted residuals vs. population-predicted concentrations, individual-predicted vs. observed concentrations, and population-weighted residuals vs. time. In the development of the model, covariates were added into or removed from a model based on likelihood ratio tests at a significance level of 5 % for forward inclusion and 1 % for backward elimination.

### Development of the three-compartment model

PK data from both the IV and oral routes were fitted simultaneously in order to allow for estimation of the oral bioavailability. Several models were explored, including two-compartment and three-compartment models with first-order elimination. A three-compartment model was adopted with first-order input (in case of oral formulation) using the following PK parameters: CL and volume of the central compartment (V2), absorption rate constant (*K*_*a*_), bioavailability (*F*), and rate constants between the central and two peripheral compartments: *k*_23_, *k*_32_, *k*_24_, and *k*_42_. A combined additive and proportional residual error model was selected.

### Analysis of the effects of covariates

The following covariates were investigated for their relationship to panobinostat PK for CL and V2: race (Caucasian, black, Asian, and other), weight, body mass index, BSA, sex, age, CrCL, tumor type, and liver status, which was categorized as normal, mild, moderate, or severe according to the following definitions (total bilirubin (TB), aspartate aminotransferase (AST), upper limit of normal (ULN)):

$$ \begin{array}{l}\mathrm{N}\mathrm{or}\mathrm{mal}\ \left(\mathrm{T}\mathrm{B}\ \le\ \mathrm{U}\mathrm{L}\mathrm{N},\ \mathrm{A}\mathrm{S}\mathrm{T}\ \le\ \mathrm{U}\mathrm{L}\mathrm{N}\right)\hfill \\ {}\mathrm{Mild}\ \left(\left(\mathrm{T}\mathrm{B}\ \le\ \mathrm{U}\mathrm{L}\mathrm{N}\ \mathrm{and}\ \mathrm{A}\mathrm{S}\mathrm{T}>\mathrm{U}\mathrm{L}\mathrm{N}\right),\ \mathrm{or}\ \left(\mathrm{U}\mathrm{L}\mathrm{N}\ \le\ \mathrm{T}\mathrm{B}\ \le\ 1.5\ \mathrm{U}\mathrm{L}\mathrm{N}\right)\right)\hfill \\ {}\mathrm{Moderate}\left(1.5\ \mathrm{U}\mathrm{L}\mathrm{N}\ \le\ \mathrm{T}\mathrm{B}\ \le\ 3\ \mathrm{U}\mathrm{L}\mathrm{N}\right)\hfill \\ {}\mathrm{S}\mathrm{evere}\ \left(3\ \mathrm{U}\mathrm{L}\mathrm{N}<\mathrm{T}\mathrm{B}\right)\hfill \end{array} $$

The effect of concomitant medications was also investigated for panobinostat CL, with the following groups included in the analysis: drugs known to prolong QT, CYP2D6 substrates, strong CYP3A4/5 inhibitors, CYP3A4 inducers, and sensitive CYP3A4 substrates. The effects of the CSF vs. FMI formulations on *F* and *K*_*a*_ were also analyzed.

For CL and V2, covariates (*x*_*i*_) and random effects (*η*_CL_, *η*_*V*_) were incorporated into NONMEM models using the following method. A reference value of *x*_*i*_, denoted by *x*_*i*0_, was selected; for continuous covariates, the median value was the reference. Dichotomous variables were assigned a numerical value of 0 for the reference category or 1 for the other category. In general, categorical variables with *k* categories were assigned *k*-1 0-or-1 variables *x*_*i*,1_, …, *x*_*i*,*k*-1_. Using this notation, *x*_*i*_ was modeled as affecting, e.g., CL as follows:

$$ \mathrm{C}\mathrm{L}=\theta 1\times \cdots \times {z}_{il}\times \cdots \times \exp \left({\eta}_{\mathrm{CL}}\right) $$

where

$$ {z}_{ij}={\left({x}_i/{x}_{io}\right)}^{\theta_{ij}} $$

if *x*_*i*_ was continuous, and

$$ {z}_{ij}={\theta}_{ij}^{x_i} $$

if *x*_*i*_ was dichotomous, and

$$ {z}_{ij}={\theta}_{ij,1}^{x_i,1}\times \dots \times {\theta}_{ij,k-1}^{x_{i,k-1}} $$

if *x*_*i*_ was categorical with *k* categories.

The *z*_*ij*_ terms were tested for inclusion in models using a forward selection procedure for entering *z*_*ij*_. A covariate was either included on or excluded from both CL and V2. First, the models with exactly one *z*_*i*1_ and exactly one *z*_*i*2_ entered into the model were fitted. The (*z*_*i*1_ and *z*_*i*2_) pair with the smallest *P* < 0.05 based on the likelihood ratio test was selected. Next, the full model with all the covariates chosen at the first step was fitted. Finally, only those covariate effects that had statistically significant estimations at the 1 % level were kept in the final model.

The random effects *η*_CL_ and *η*_*V*_ were modeled as normally distributed with mean zero and with a full 2 × 2 covariance matrix.

### Assessment of predictive performance

To validate the final model, predictions from the model were compared with the data using the visual predictive checks [21]. Model development used data from 14 studies with heterogeneous study designs. For the validation, a subset of six studies that had similar PK sampling designs was selected. Another criterion for inclusion was that the study evaluated the FMI formulation at a dose regimen of 20 mg per day on days 1, 3, and 5 each week. From such studies, only the 20 mg regimen was used because it is the recommended clinical dose regimen for multiple myeloma. One study that evaluated the CSF, B1101, was also included because this study was conducted in 13 Japanese patients, and comparison of Japanese and Western patients was of interest. For study B1101, only observations from the 20 mg cohort were used in the assessment.

The predictive assessment focused on day 1 (postdose: 15 min, 1–2 h, 3–4 h) and day 8 (postdose: 15 min, 1–2 h, 3–4 h) dosing. For B1101, day 15 was used instead of day 8, which was not available; it was expected that the profile would be similar because the half-life of panobinostat is 16 h; therefore, samples would be collected after steady state.

Data were simulated according to the design by which the observed data were collected; 300 replicates of the original data were simulated. The raw data were plotted vs. time along with the 10th and 90th percentiles of observed values within specified intervals; the percentiles of the simulated data within those intervals were then superimposed.

## Results

### Patients

**Fig. 1:**
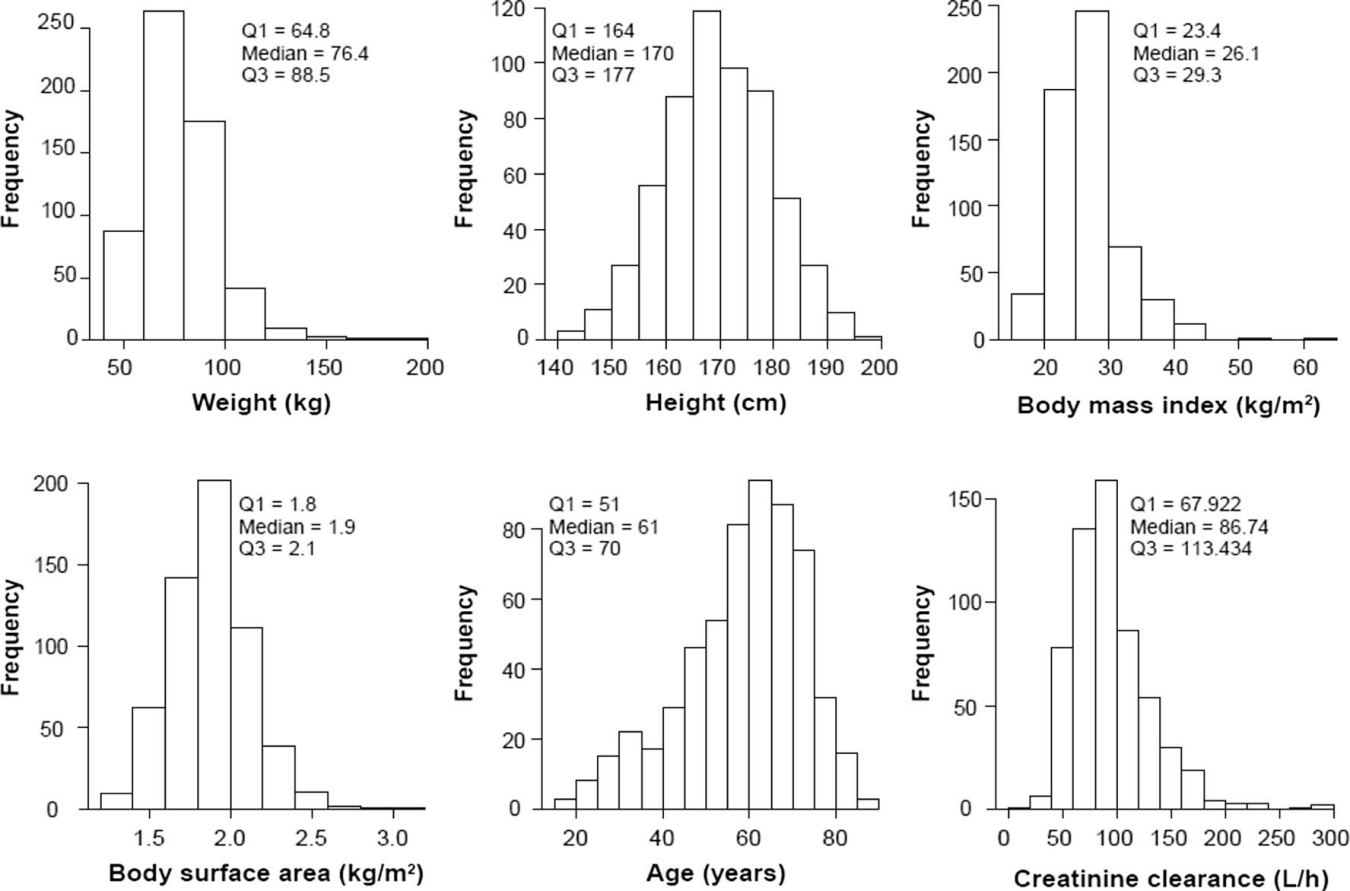
Histograms and summary statistics of baseline covariates for the 581 patients in the analysis data set

PK data were available for 7834 panobinostat concentration-time points from 581 patients who received panobinostat in one of 14 open-label phase 1 or phase 2 studies. Details about the patient demographics in each of these studies are presented in Table 1 and Fig. 1. Eighty-seven patients participated in studies investigating IV panobinostat, whereas 494 patients received oral panobinostat. Of these 494 patients, 106 patients received the CSF, and 388 patients received the FMI oral formulation.

As shown in Table 1 and Fig. 1, there were more men than women (362 vs. 219, respectively). The median age of the PK population was 61 years (range, 16–88 years), the median weight was 76.4 kg (range, 41–196.4 kg), and the median height was 170 cm (range, 143–198 cm). The vast majority of patients were Caucasian (*n* = 496), followed by black (*n* = 34) and Asian (*n* = 27). The remaining 24 patients were classified as “other.”

### Pharmacokinetics and base model

**Fig. 2:**
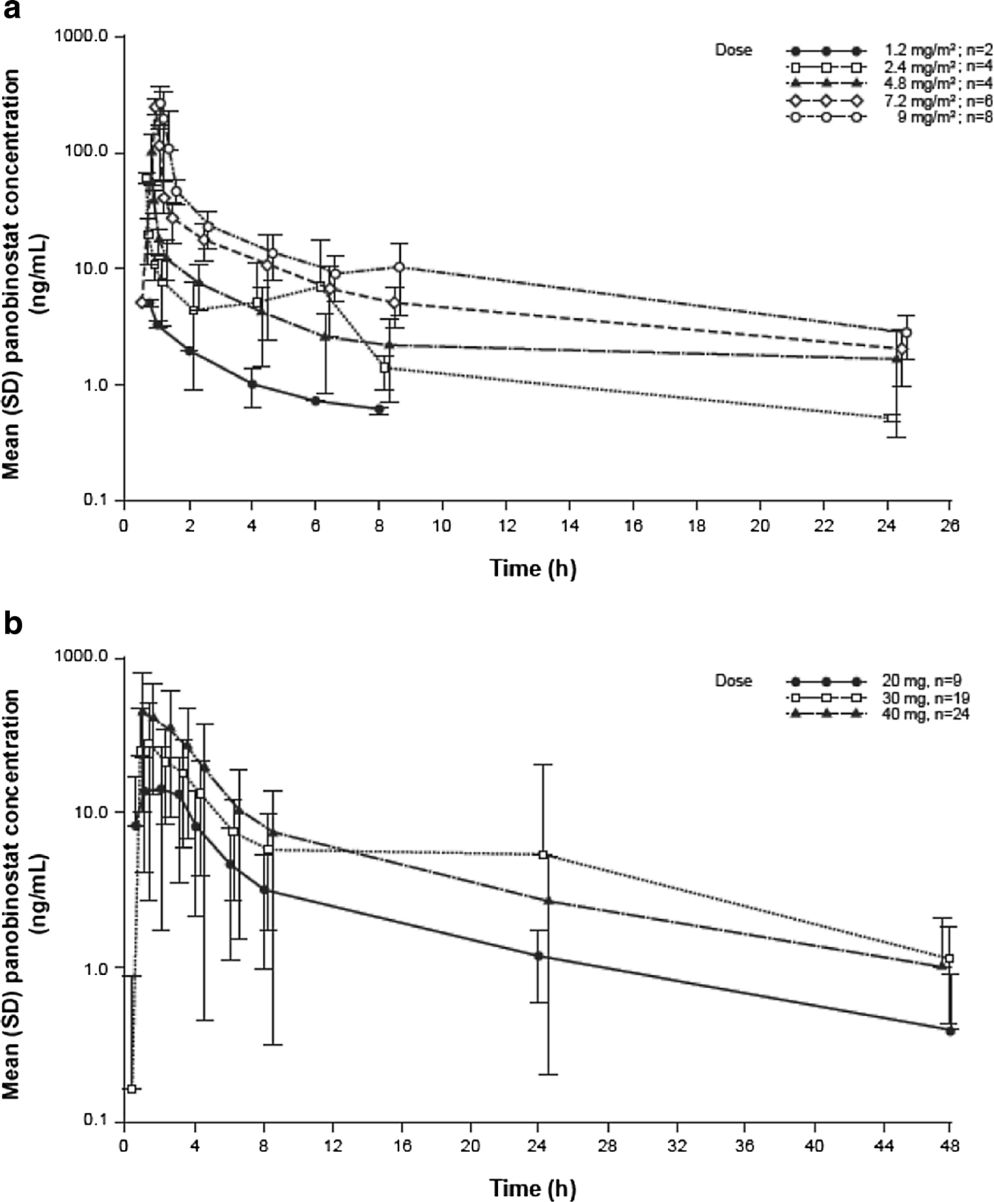
Arithmetic mean (SD) plasma panobinostat concentration-time plots following day 1 for intravenous panobinostat at doses of 1.2, 2.4, 4.8, 7.2, and 9 mg/m^2^ (**a**) and oral panobinostat at doses of 20, 30, and 40 mg for all schedules (**b**). Semilogarithmic view (data on file)

The plasma vs. time profile of panobinostat exhibited a tri-exponential disposition profile. This is illustrated by representative plots for the IV formulation by data from the A2101 study (Fig. 2a) and the oral formulation from the B2102 study (Fig. 2b) [3, 10].

The results of a multiplicative model that related CL and V2 of panobinostat to dose did not produce a significant reduction in the OFV. Therefore, the PK of panobinostat was determined to be dose proportional over the dose range of 10 to 80 mg across the 14 studies.

Following the evaluation of several structural models, a linear three-compartment base model with first-order absorption and combined additive and proportional residual error was found to best describe the disposition of panobinostat. Fluxes of drug mass between the central and peripheral compartments were parameterized in terms of rate constants *K*_23_, *K*_32_, *K*_24_, and *K*_42_ in units of hours^−1^.

### Final models

Following the methodology described previously, covariate analysis led to a final model where CL and V2 depended on BSA, age, and race, and where absorption rate depended on formulation.

In response to suggestions from reviewers, additional model development was undertaken and summarized in Table 2. A second final model was determined wherein a formulation-dependent lag was added to the absorption process; the intercompartmental rate constants were replaced by intercompartmental clearances and peripheral volumes, which were associated with multiplicative random effects; BSA was removed from the model, and all clearances were assumed proportional to weight^0.75^ and all volumes to weight^1^ [22]; and age effects were associated with the intercompartmental clearances and peripheral volumes.

**Table 2:** Model development

Model	Number of parameters	NONMEM objective	AIC	BIC
0. Base: Three-compartment, linear model with first-order absorption, parameterized in terms of *K*_*a*_, CL, V2, *K*_23_, *K*_32_, *K*_24_, *K*_42_; IIV on CL and V2; combined additive and proportional residual error	14	33830	33858	33885
1. Final 1: Add dependence on BSA, age, and race to CL and V2; add dependence on formulation to *K*_a_	24	33758	33806	33851
2. Add formulation-dependent absorption lag	26	33698	33750	33799
3. Change parameterization to *K*_a_, CL, V2, Q3, V3, Q4, V4; add IIV to Q3, V3, Q4, V4; replace dependence of CL and V2 on BSA with allometric dependence of all clearances and volumes on weight	30	32616	32676	32733
4. Final 2: Add dependence on age to Q3, V3, Q4, V4.	34	32591	32659	32723

Each step of model development in Table 2 significantly improved on the previous in terms of AIC and BIC, but the models labeled 3 and 4 (the latter being the second final model) did not satisfy NONMEM’s default convergence criterion.

Parameter estimates from the two final models with bootstrap confidence intervals can be found in the Supplementary Material. With the first final model, the absolute bioavailability of oral panobinostat was estimated as 21.4 %. The estimated median CL was 33.1 L/h for a 61-year-old Caucasian patient with BSA 1.9 m^2^. The estimated inter-patient variation in CL was large, 74 %. Results were similar for the second final model, and more generally, estimates of shared parameters were similar between the two final models (see Supplementary Material).

**Fig. 3:**
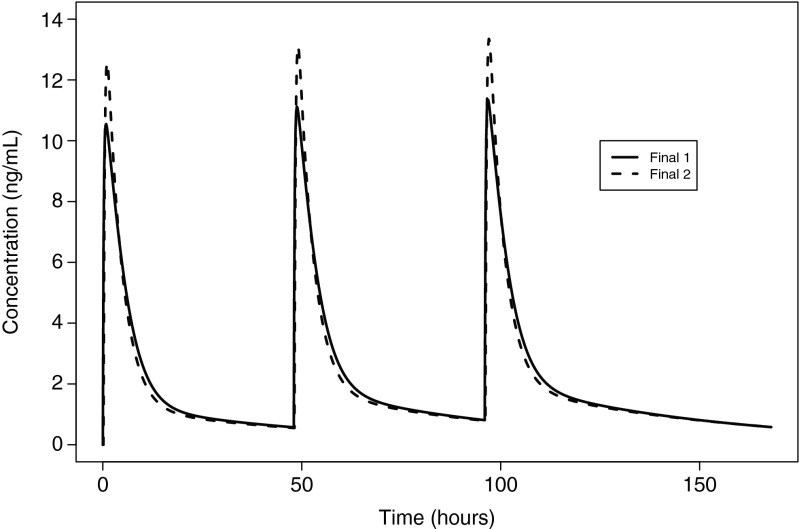
Simulated concentration time curves for the two final models, for a 61-year-old Caucasian patient with BSA 1.9 m^2^ (first final model) or weight 76.4 kg (second final model) receiving 20 mg of the FMI on days 1, 3, and 5

Figure 3 displays simulated concentrations vs. time for a typical patient given 20 mg of the FMI on Monday, Wednesday, and Friday. The two final models predict similar profiles except for the second’s higher peak. Supplementary Table S4 demonstrates further similarity with respect to the predictions that account for inter-patient variability.

Supplementary Figures S1 and S2 display goodness-of-fit plots for the final models. The models were generally adequate in describing the heterogeneous data set. Only 27 plasma panobinostat concentrations from 25 patients had predictions that exhibited a significant lack of fit (absolute value of conditional weighted residual >4) for the first final model (23 observations from 22 patients for the second).

**Fig. 4:**
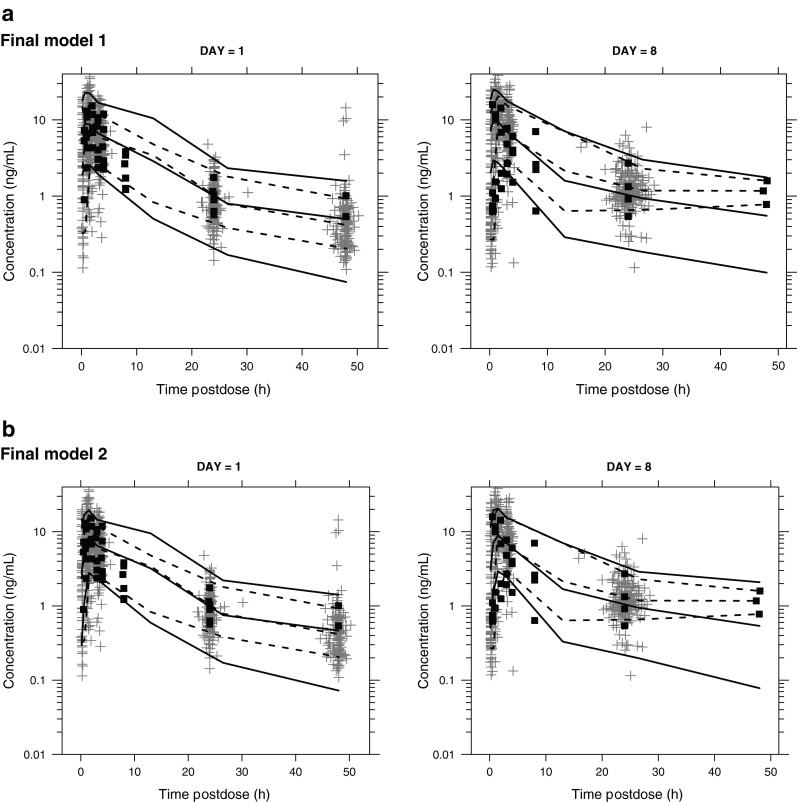
**a**–**b** Visual predictive checks. Data for non-Japanese patients are indicated by *gray plus sign*s. Data for Japanese patients are indicated by *black squares*. The *dashed lines* connect the medians and the 10th and 90th percentiles of the raw data in the defined bins. The *solid lines* connect the medians and the 10th and 90th percentiles of the simulations. Dosing was 20 mg on days 1, 3, 5, and 8

Figure 4 displays the predictive checks. A total of 1393 PK observations from patients across six studies receiving 20 mg Monday, Wednesday, and Friday were included. The models capture the trend in the data, although they predict more variability at and beyond 24 h postdose. The second final model, with an absorption lag, does somewhat better in the absorption phase. Although overall numbers were small, there were no clear inadequacies of the model with respect to the Japanese patients.

### Covariate analysis

Following the original methodology, the first final model included BSA, age, and race as covariates affecting CL and V2. In addition, the formulation (CSF vs. FMI) had a significant impact on the *K*_*a*_ (but not on the bioavailability) of oral panobinostat. None of the other clinical covariates investigated (route of administration, creatinine clearance at baseline, indices of liver status, and concomitant medications) showed a statistically significant effect on panobinostat PK. In addition, route of administration (IV vs. oral) did not show a statistically significant effect on CL or V2.

In the second final model, BSA was replaced by weight, which was also applied to intercompartmental clearances and peripheral volumes, as was age.

Table 3 summarizes the effects of covariates by showing typical values (i.e., with random effects set to zero) of AUC, *C*_max_, and the trough concentration over 48 h following a 20-mg dose for various combinations of covariates. According to the first final model, for Caucasian patients of median age 61 years, AUC decreased from 104 to 88 ng· h/mL as BSA increased from the first to third quartiles, 1.8 to 2.1 m^2^. For Caucasian patients of median BSA 1.9 m^2^, AUC decreased from 102 to 95 ng· h/mL as age increased from the first to third quartiles, 51 to 70 years. For patients of median BSA and median age, AUC ranged across the four race categories from 80 to 116 ng· h/mL. The variation across covariate groups was not large compared to unexplained variation induced by random effects. As shown in Supplementary Table S4, when 300 Caucasian patients of median age and BSA were simulated with random effects, the interquartile range of AUC0-48 h was 61–145 ng h/mL. Results were similar for the second final model.

**Table 3:** Model-predicted variation in panobinostat exposure across covariates

a Final model 1					
Body surface area (m^2^)	Age (years)	Race	*C*_max_, 0–48 h (ng/mL)	C48 h (ng/mL)	AUC, 0–48 h (ng·h/mL)
1.9	61	Caucasian	10.6	0.574	98.1
1.8	61	Caucasian	11.3	0.601	104.1
2.1	61	Caucasian	9.3	0.526	87.8
1.9	51	Caucasian	11.2	0.583	102.3
1.9	70	Caucasian	10.1	0.566	94.9
1.9	61	Asian	8.0	0.517	79.6
1.9	61	Black	9.0	0.607	90.9
1.9	61	Other	10.4	0.893	116.3
b Final model 2					
Weight (kg)	Age (years)	Race	Cmax, 0–48 h (ng/mL)	C48h (ng/mL)	AUC,0-48h (ng∙h/mL)
76.4	61	Caucasian	12.6	0.552	94.7
64.8	61	Caucasian	14.4	0.630	108.3
88.5	61	Caucasian	11.1	0.491	84.0
76.4	51	Caucasian	13.0	0.569	98.9
76.4	70	Caucasian	12.2	0.538	91.6
76.4	61	Asian	10.1	0.433	82.3
76.4	61	Black	11.1	0.606	98.0
76.4	61	Other	14.3	0.914	123.3

## Discussion

Panobinostat is a pan-HDAC inhibitor recently approved by the FDA in combination with bortezomib and dexamethasone for the treatment of patients with multiple myeloma who have received at least two prior regimens, including bortezomib and an immunomodulatory agent [Farydak^®^ US Prescribing Information, 2015].

This is the first reported population PK study of oral and IV panobinostat, which used 7834 PK measurements from 581 patients across 14 phase 1 and phase 2 studies. Large-scale population PK analyses conducted during the development of novel chemical entities are used to guide dose optimization and administration to provide maximal benefit to the patients. The PK behavior of panobinostat was well described by a three-compartment model with first-order absorption and elimination, and the model was evaluated for its predictive performance.

Body size, age at baseline, and race were statistically significant covariates on the parameters of the model. However, due to the magnitude of these effects as compared with the unexplained interindividual variability, none of these covariates were considered to be clinically relevant in the studied population. None of the other covariates tested were found to significantly affect CL and V2. It may be noted that in dedicated studies, ketoconazole, a CYP3A4 inhibitor, and liver impairment were associated with significant increases in panobinostat exposure [14, 24]. The US prescribing information for panobinostat recommends reduced starting doses for patients with mild or moderate hepatic impairment, and for patients taking strong CYP3A inhibitors. It also recommends avoidance of strong CYP3A inducers. That comedications and liver status were not found significant here may have been related to study design (patients enrolled in these trials were usually selected to have baseline bilirubin and AST/ALT ≤1.5 and 2 × ULN, respectively, and protocols recommended to avoid the use of concomitant strong CYP3A4 inhibitors) or due to limited power given the overall high level of interindividual variability.

The current analysis also demonstrated that the absolute bioavailability associated with the FMI formulation, which is intended for commercialization, was 21 %. This is consistent with the value derived from the geometric mean ratio of the AUCs based on noncompartmental analysis (data on file). The terminal half-life calculated based on the final parameter estimates was approximately 37 h, which was similar to values found in the control group of two independent single-dose organ impairment studies in which PK sampling was carried out up to 96 h postdose [23, 24]. However, there is little accumulation with dosing every 48 h, as indicated in Fig. 3.

Based on the results of the population PK analysis, the starting dose of panobinostat does not need to be adjusted for the covariates analyzed in populations similar to those studied. Subsequent individual dose titration according to tolerability and efficacy should follow the drug prescribing information and study protocols as appropriate.

The model used for the population PK analysis has also been applied as an input to a PK/PD model for platelet response developed to better understand the relationship between thrombocytopenia, a frequent adverse event reported with panobinostat [3], and dose and schedule [24]. The PK/PD model was also used to support selection of the dose regimen for the recently completed phase 3 trial of panobinostat in combination with bortezomib and dexamethasone, which demonstrated a significant increase in median progression-free survival for patients who received panobinostat [18].

## Electronic supplementary material

ESM 1 (PDF 978 kb)

ESM 2 (PDF 99 mb)

ESM 3 (DOC 37 kb)

ESM 4 (DOC 26 kb)

ESM 5 (DOC 56 kb)

ESM 6 (DOC 26 kb)

ESM 7 (DOC 71 kb)

ESM 8 (DOC 40 kb)
