# Supplementary figures and images for: Population pharmacokinetics of intravenous and oral panobinostat in patients with hematologic and solid tumors
Source: Eur J Clin Pharmacol. 2015 May 5;71(6):663–72. doi: 10.1007/s00228-015-1846-7 (PMC4430599; doi:10.1007/s00228-015-1846-7)

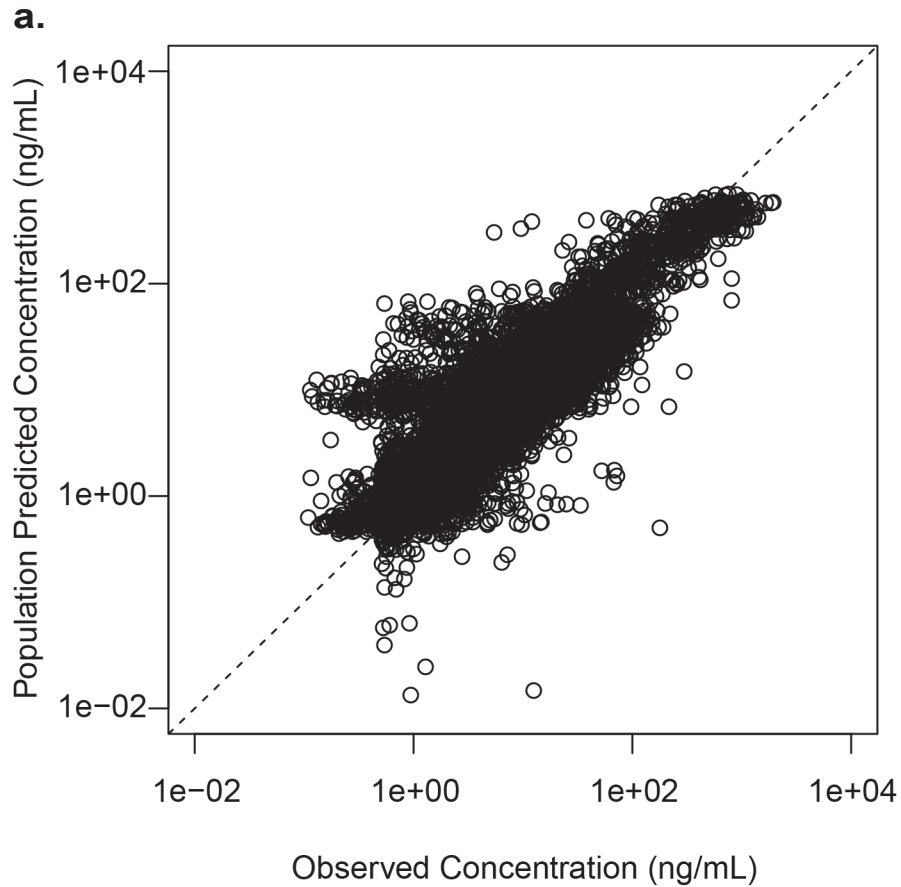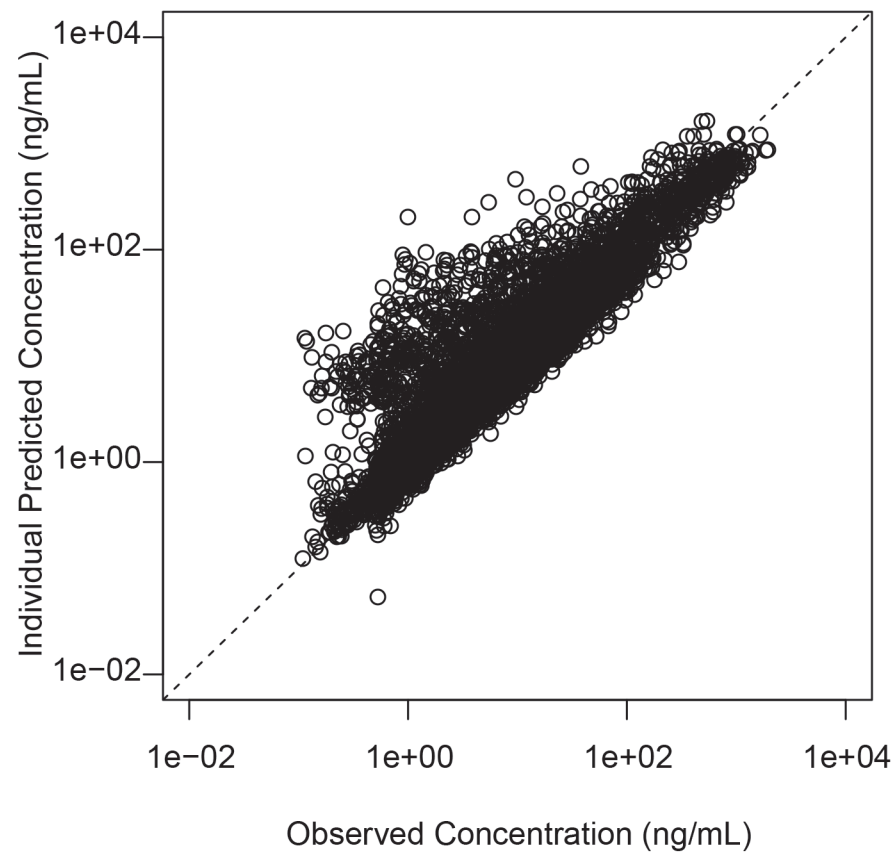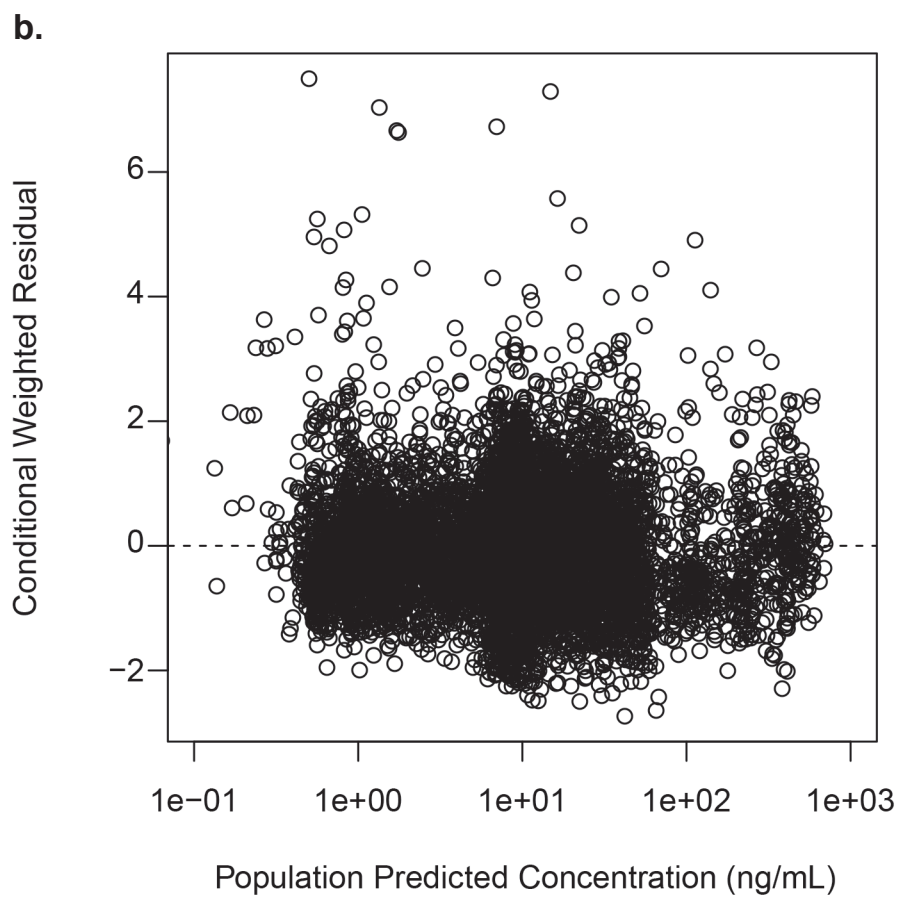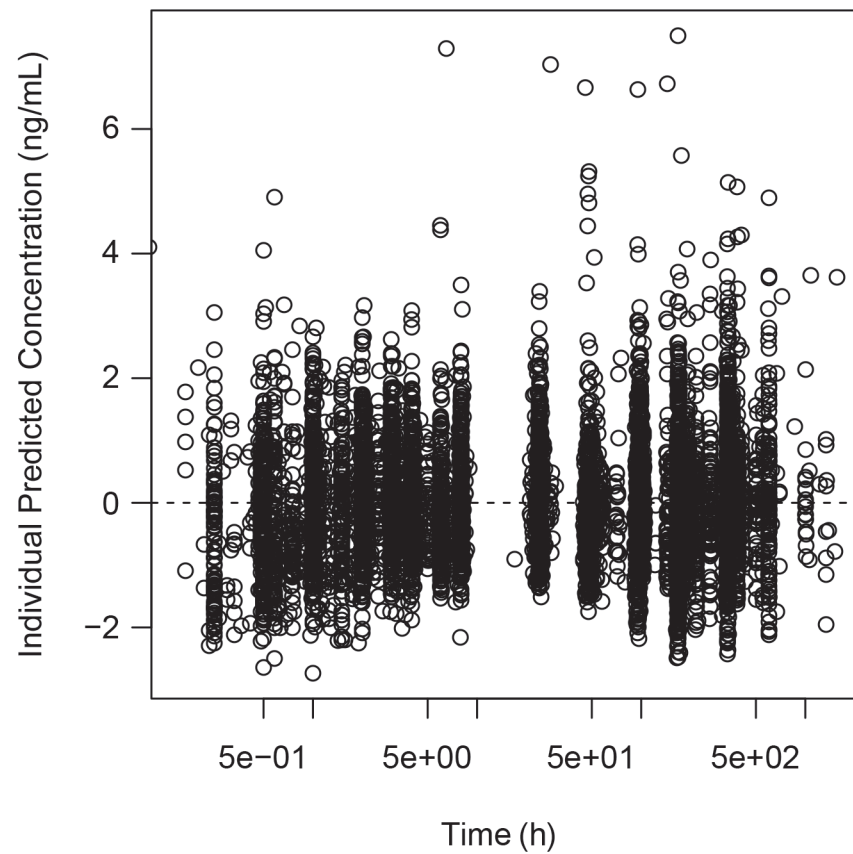

Supplement: Supplementary file 1 — (PDF 978 kb) [file 228_2015_1846_MOESM1_ESM.pdf]

**a.**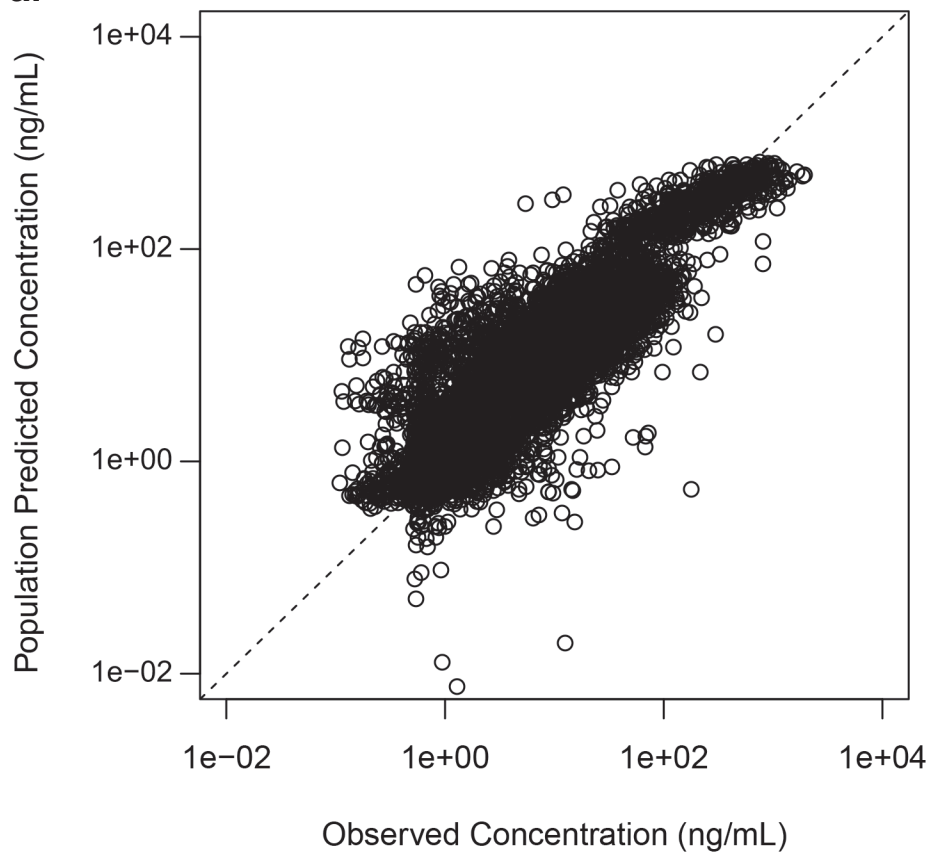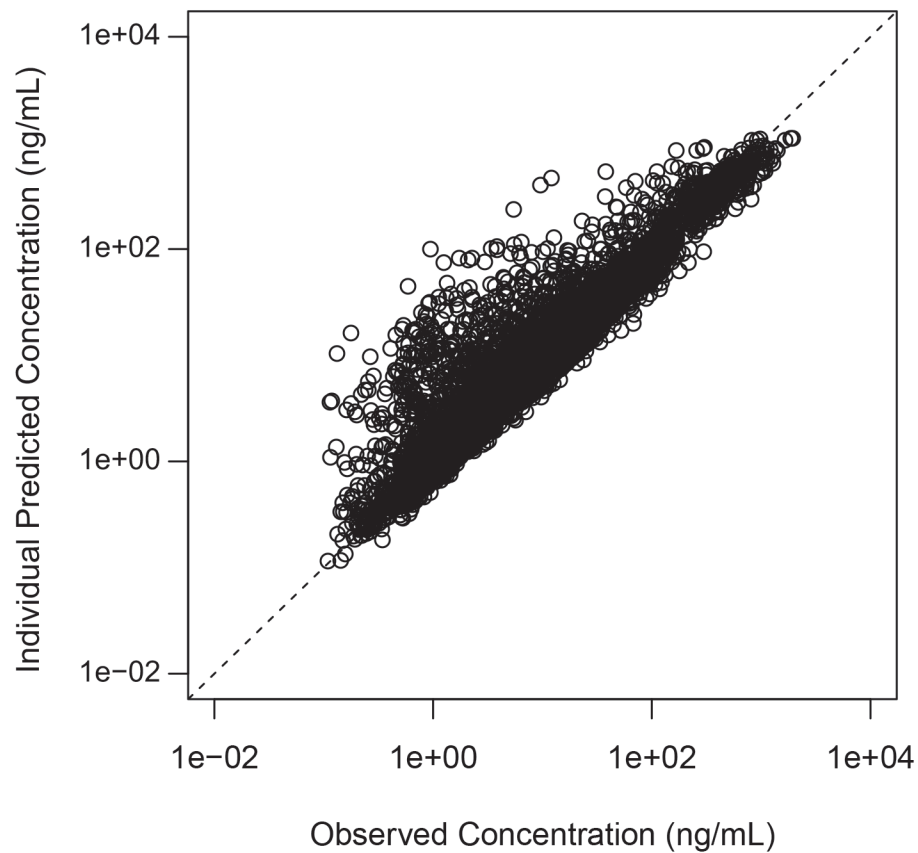**b.**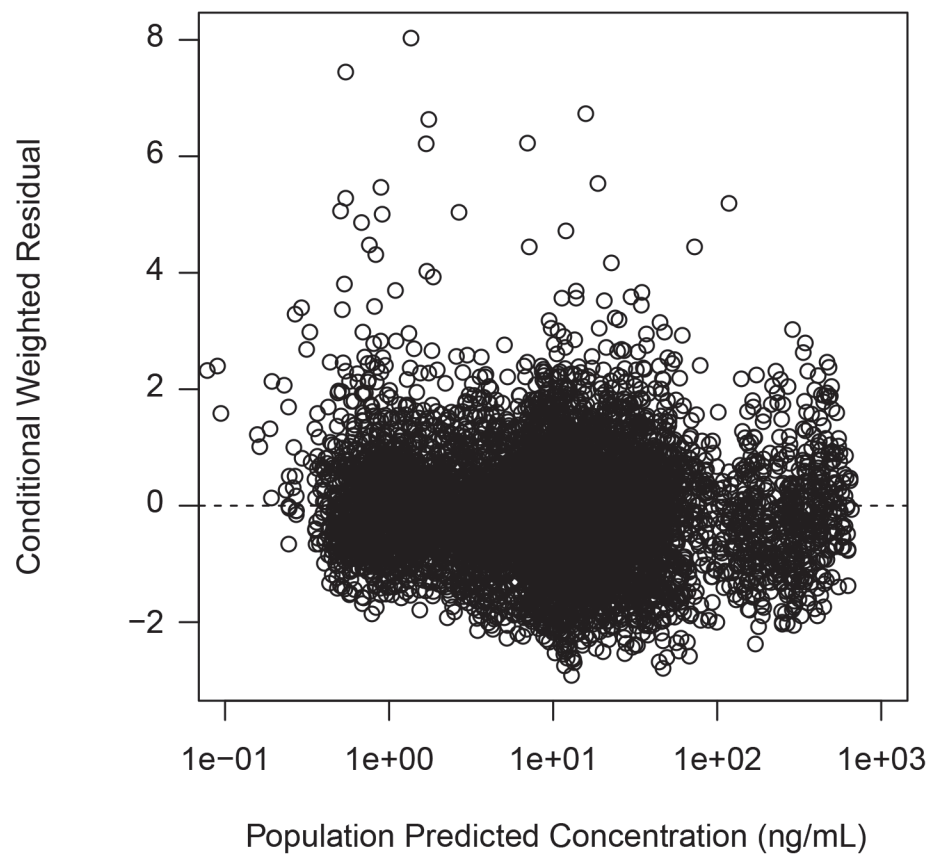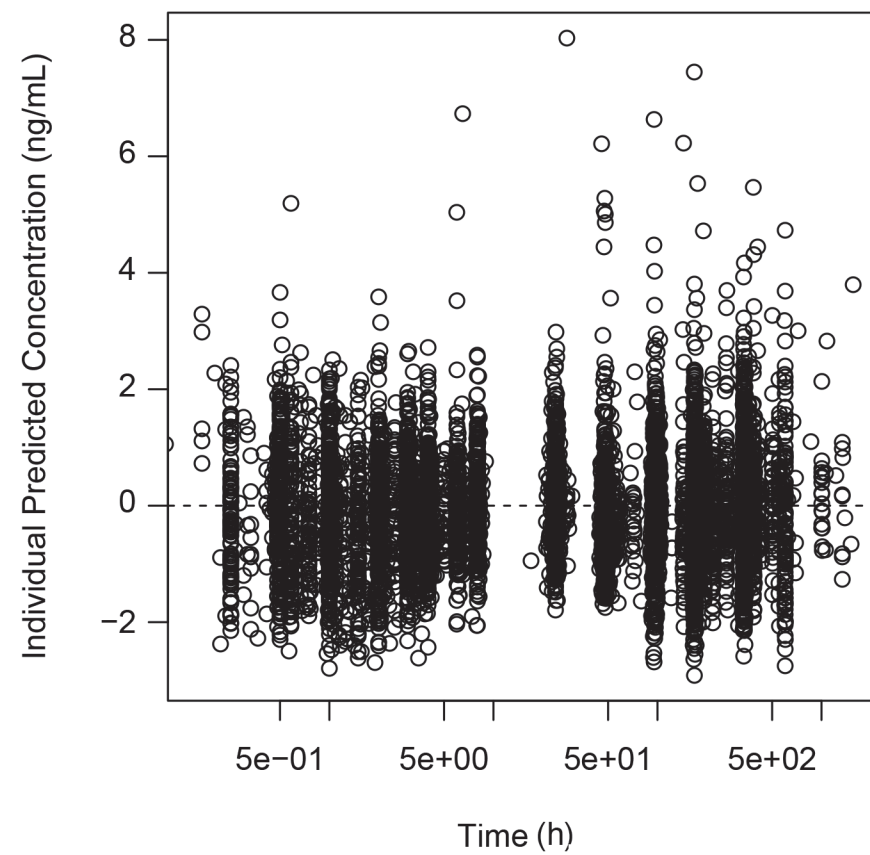

Supplement: Supplementary file 2 — (PDF 99 mb) [file 228_2015_1846_MOESM2_ESM.pdf]
