# Supplementary material for: Population pharmacokinetics of intravenous and oral panobinostat in patients with hematologic and solid tumors
Source: Eur J Clin Pharmacol. 2015 May 5;71(6):663–72. doi: 10.1007/s00228-015-1846-7 (PMC4430599; doi:10.1007/s00228-015-1846-7)
Supplement: Supplementary file 3 — (DOC 37 kb) [file 228_2015_1846_MOESM3_ESM.doc]

**Supplementary Material**

**Table S1 Studies included in the analysis**

| Study | N | Population |
| --- | --- | --- |
| A2101 [10] | 72 | Phase 1A, 4-arm, multicenter, dose-escalation study of patients with advanced solid tumors, HL, or NHL |
| A2102 [6] | 15 | Phase 1A/2, 2-arm, dose-escalation study in patients with advanced hematologic malignancies |
| B1101^a^ [12] | 13 | Phase 1, open-label, dose-escalation study in Japanese patients |
| B1201^a^ [11] | 4 | Phase 2, open-label, multicenter (Japan) study in patients with CTCL and T-cell lymphoma |
| B2101 [13] | 93 | Phase 1A, 6-arm dose-escalation study in patients with advanced solid tumor or NHL |
| B2102 [3] | 140 | A phase 1A/2, 2-arm, multicenter, open-label, dose-escalation study in adult patients with advanced hematologic malignancies |
| B2109 [5] | 16 | Phase 1, open-label, multicenter, DDI study with a sensitive CYP2D6 substrate in advanced solid tumors |
| B2110 [14] | 14 | Phase 1, open-label, multicenter, DDI study with a strong CYP3A4 inhibitor in advanced solid tumors |
| B2111 [15] | 10 | Phase 1, open-label, multicenter, food effect (fasting and fed conditions) in advanced solid tumors |
| B2201^a^ [4] | 120 | Phase 2 study in adult patients with refractory CTCL |
| B2202^a^ [9] | 22 | Phase 2, single-arm, multicenter, international study in patients with CML with resistant disease |
| B2203^a^ [8] | 31 | Phase 2 single-arm, multicenter, international study in patients with refractory MM who have received ≥ 2 prior lines of therapy |
| B2211^a^ [7] | 17 | Phase 2, single-arm, multicenter, international study in patients with accelerated phase or blast crisis CML with resistant disease |
| E2214 [16] | 14 | Phase 2, open-label, multicenter in relapsed/refractory HL |
| Total | 581 |  |

###### Abbreviations: BSA, body surface area; CML, chronic myeloid leukemia; CSF, clinical service formulation; CTCL, cutaneous T-cell lymphoma; DDI, drug-drug interaction; FMI, final market image; HL, Hodgkin lymphoma; IV, intravenous; MM, multiple myeloma; NHL, non-Hodgkin lymphoma.

^a^ Patients from these studies on 20 mg FMI (or CSF, in B1101) were included in the analysis of predictive performance.
