# Supplementary material for: Population pharmacokinetics of intravenous and oral panobinostat in patients with hematologic and solid tumors
Source: Eur J Clin Pharmacol. 2015 May 5;71(6):663–72. doi: 10.1007/s00228-015-1846-7 (PMC4430599; doi:10.1007/s00228-015-1846-7)
Supplement: Supplementary file 4 — (DOC 26 kb) [file 228_2015_1846_MOESM4_ESM.doc]

**Table S2a: $PK and $ERROR from the first final model**

$PK

AS=0

BL=0

OT=0

IF (RACE .EQ. 3) AS=1

IF (RACE .EQ. 2) BL=1

IF (RACE .EQ. 88) OT=1

CL1=THETA(1)*EXP(ETA(1))*(BSA0/1.9)**THETA(10)

CL=CL1*(AGE0/61)**THETA(12)*THETA(14)**(AS)*THETA(16)**(BL)*THETA(18)**(OT)

V21=THETA(2)*EXP(ETA(2))*(BSA0/1.9)**THETA(11)

V2=V21*(AGE0/61)**THETA(13)*THETA(15)**(AS)*THETA(17)**(BL)*THETA(19)**(OT)

K = CL/V2

K23=THETA(3)

K32=THETA(4)

K24=THETA(5)

K42=THETA(6)

IF (IV.EQ.1) THEN

KA=0

ELSE

KA=THETA(7)*(1-FORM) +THETA(8)*FORM

ENDIF

IF (IV.EQ.1) THEN

TVF1=1

ELSE

TVF1=THETA(9)

ENDIF

F1=TVF1

D2=DUR

S2=V2/1000

$ERROR

IPRED=F

Y=F*(1+EPS(1))+EPS(2)
