# Supplementary material for: Population pharmacokinetics of intravenous and oral panobinostat in patients with hematologic and solid tumors
Source: Eur J Clin Pharmacol. 2015 May 5;71(6):663–72. doi: 10.1007/s00228-015-1846-7 (PMC4430599; doi:10.1007/s00228-015-1846-7)
Supplement: Supplementary file 5 — (DOC 56 kb) [file 228_2015_1846_MOESM5_ESM.doc]

Table S2b: Parameter estimates from the first final model

| NONMEM Name | Interpretive Name (Units) | Estimate | Bootstrap Results | | | |
| --- | --- | --- | --- | --- | --- | --- |
| Theta |  |  | SE | PctSE | Q10 | Q90 |
| 1 | CL (L/h) | 33.085 | 2.225 | 6.704 | 30.467 | 36.286 |
| 2 | V2 (L) | 24.838 | 2.447 | 9.836 | 21.843 | 27.863 |
| 3 | K23 (1/h) | 1.810 | 0.193 | 10.582 | 1.584 | 2.070 |
| 4 | K32 (1/h) | 0.507 | 0.039 | 7.633 | 0.464 | 0.559 |
| 5 | K24 (1/h) | 1.424 | 0.134 | 9.372 | 1.263 | 1.610 |
| 6 | K42 (1/h) | 0.040 | 0.002 | 4.751 | 0.037 | 0.042 |
| 7 | KA.FMI (1/h) | 0.321 | 0.027 | 8.490 | 0.285 | 0.354 |
| 8 | KA.CSF (1/h) | 0.544 | 0.025 | 4.529 | 0.515 | 0.578 |
| 9 | F1 | 0.214 | 0.015 | 6.902 | 0.195 | 0.235 |
| 10 | CL.BSA | 1.002 | 0.228 | 22.804 | 0.724 | 1.319 |
| 11 | V2.BSA | 1.359 | 0.193 | 14.499 | 1.068 | 1.576 |
| 12 | CL.AGE | 0.176 | 0.093 | 56.049 | 0.047 | 0.280 |
| 13 | V2.AGE | 0.396 | 0.091 | 23.558 | 0.270 | 0.499 |
| 14 | CL.ASIAN | 1.171 | 0.107 | 9.157 | 1.047 | 1.313 |
| 15 | V2.ASIAN | 1.373 | 0.177 | 12.744 | 1.169 | 1.609 |
| 16 | CL.BLACK | 1.010 | 0.143 | 14.061 | 0.854 | 1.195 |
| 17 | V2.BLACK | 1.241 | 0.196 | 15.596 | 1.017 | 1.490 |
| 18 | CL.OTHER | 0.719 | 0.135 | 18.580 | 0.559 | 0.908 |
| 19 | V2.OTHER | 1.127 | 0.144 | 12.689 | 0.954 | 1.324 |
| Sigma |  |  |  |  |  |  |
| 1 | VAR.PROP | 0.242 | 0.011 | 4.450 | 0.228 | 0.255 |
| 2 | VAR.ADD | 0.013 | 0.009 | 80.332 | 0.000 | 0.021 |
| Omega |  |  |  |  |  |  |
| 1,1 | OM.CL | 0.439 | 0.053 | 12.391 | 0.368 | 0.501 |
| 2,1 | OM.CLV2 | 0.178 | 0.021 | 12.277 | 0.146 | 0.201 |
| 2,2 | OM.V2 | 0.334 | 0.029 | 8.746 | 0.295 | 0.367 |
