# Supplementary material for: Population pharmacokinetics of intravenous and oral panobinostat in patients with hematologic and solid tumors
Source: Eur J Clin Pharmacol. 2015 May 5;71(6):663–72. doi: 10.1007/s00228-015-1846-7 (PMC4430599; doi:10.1007/s00228-015-1846-7)
Supplement: Supplementary file 6 — (DOC 26 kb) [file 228_2015_1846_MOESM6_ESM.doc]

**Table S3a: $PK and $ERROR from the second final model**

AS=0

BL=0

OT=0

IF (RACE .EQ. 3) AS=1

IF (RACE .EQ. 2) BL=1

IF (RACE .EQ. 88) OT=1

CL1=THETA(1)*EXP(ETA(1))*(WT0/70)**THETA(10)

CL=CL1*(AGE0/61)**THETA(12)*THETA(14)**(AS)*THETA(16)**(BL)*THETA(18)**(OT)

V21=THETA(2)*EXP(ETA(2))*(WT0/70)**THETA(11)

V2=V21*(AGE0/61)**THETA(13)*THETA(15)**(AS)*THETA(17)**(BL)*THETA(19)**(OT)

K = CL/V2

Q3=THETA(3)*(WT0/70)**0.75*EXP(ETA(3))*(AGE0/61)**THETA(22)

V3=THETA(4)*(WT0/70)*EXP(ETA(4))*(AGE0/61)**THETA(23)

Q4=THETA(5)*(WT0/70)**0.75*EXP(ETA(5))*(AGE0/61)**THETA(24)

V4=THETA(6)*(WT0/70)*EXP(ETA(6))*(AGE0/61)**THETA(25)

IF (IV.EQ.1) THEN

KA=0

ALAG1=0

ELSE

KA=THETA(7)*(1-FORM) +THETA(8)*FORM

ALAG1=THETA(20)*(1-FORM) + THETA(21)*FORM

ENDIF

IF (IV.EQ.1) THEN

TVF1=1

ELSE

TVF1=THETA(9)

ENDIF

F1=TVF1

D2=DUR

S2=V2/1000

$ERROR

IPRED=F

Y=F*(1+EPS(1))+EPS(2)
