# Supplementary material for: Population pharmacokinetics of intravenous and oral panobinostat in patients with hematologic and solid tumors
Source: Eur J Clin Pharmacol. 2015 May 5;71(6):663–72. doi: 10.1007/s00228-015-1846-7 (PMC4430599; doi:10.1007/s00228-015-1846-7)
Supplement: Supplementary file 7 — (DOC 71 kb) [file 228_2015_1846_MOESM7_ESM.doc]

Table S3b: Parameter estimates from the second final model

| NONMEM  Name | Interpretive Name (Units) | Estimate | Bootstrap Results | | | |
| --- | --- | --- | --- | --- | --- | --- |
| Theta |  |  | SE | PctSE | Q10 | Q90 |
| 1 | CL (L/h) | 28.833 | 1.654 | 5.717 | 27.046 | 31.184 |
| 2 | V2 (L) | 30.862 | 2.861 | 9.390 | 26.869 | 33.765 |
| 3 | Q3 (L/h) | 32.751 | 3.086 | 9.455 | 28.644 | 36.585 |
| 4 | V3 (L) | 71.874 | 9.204 | 12.692 | 61.651 | 83.481 |
| 5 | Q4 (L/h) | 31.088 | 2.219 | 7.228 | 28.027 | 33.405 |
| 6 | V4 (L) | 803.193 | 49.207 | 6.171 | 728.986 | 861.162 |
| 7 | KA.FMI (1/h) | 0.420 | 0.032 | 7.646 | 0.375 | 0.457 |
| 8 | KA.CSF (1/h) | 0.631 | 0.038 | 6.018 | 0.584 | 0.682 |
| 9 | F1 | 0.194 | 0.011 | 5.617 | 0.181 | 0.209 |
| 10 | CL.WT | 0.750 | NA | NA | NA | NA |
| 11 | V2.WT | 1.000 | NA | NA | NA | NA |
| 12 | CL.AGE | 0.137 | 0.089 | 63.060 | 0.018 | 0.243 |
| 13 | V2.AGE | -0.005 | 0.219 | 213.481 | -0.146 | 0.410 |
| 14 | CL.ASIAN | 1.203 | 0.101 | 8.435 | 1.087 | 1.333 |
| 15 | V2.ASIAN | 2.060 | 0.626 | 30.749 | 1.253 | 2.817 |
| 16 | CL.BLACK | 0.941 | 0.137 | 14.305 | 0.802 | 1.125 |
| 17 | V2.BLACK | 1.817 | 0.717 | 36.952 | 1.080 | 2.928 |
| 18 | CL.OTHER | 0.665 | 0.143 | 20.981 | 0.513 | 0.877 |
| 19 | V2.OTHER | 0.835 | 0.293 | 30.763 | 0.609 | 1.300 |
| 20 | LAG.FMI (h) | 0.162 | 0.009 | 5.805 | 0.158 | 0.164 |
| 21 | LAG.CSF (h) | 0.296 | 0.029 | 9.253 | 0.285 | 0.353 |
| 22 | Q3.AGE | 0.410 | 0.248 | 59.425 | 0.098 | 0.742 |
| 23 | V3.AGE | 0.713 | 0.326 | 43.368 | 0.395 | 1.145 |
| 24 | Q4.AGE | 0.212 | 0.140 | 78.251 | 0.008 | 0.345 |
| 25 | V4.AGE | 0.530 | 0.146 | 28.495 | 0.331 | 0.698 |
| Sigma |  |  |  |  |  |  |
| 1 | VAR.PROP | 0.180 | 0.008 | 4.522 | 0.169 | 0.189 |
| 2 | VAR.ADD | 0.011 | 0.005 | 46.224 | 0.001 | 0.016 |
| Omega |  |  |  |  |  |  |
| 1,1 | OM.CL | 0.407 | 0.050 | 11.790 | 0.368 | 0.487 |
| 2,1 | OM.CLV2 | 0.151 | 0.066 | 43.720 | 0.062 | 0.228 |
| 2,2 | OM.V2 | 1.668 | 0.168 | 10.033 | 1.490 | 1.902 |
| 3,3 | OM.Q3 | 0.666 | 0.163 | 23.213 | 0.532 | 0.906 |
| 4,3 | OM.Q3V3 | 0.505 | 0.181 | 32.801 | 0.354 | 0.768 |
| 4,4 | OM.V3 | 0.441 | 0.189 | 38.430 | 0.283 | 0.741 |
| 5,5 | OM.Q4 | 0.497 | 0.081 | 16.289 | 0.384 | 0.589 |
| 6,5 | OM.Q4V4 | 0.556 | 0.080 | 14.528 | 0.446 | 0.645 |
| 6,6 | OM.V4 | 0.700 | 0.113 | 16.194 | 0.559 | 0.839 |
