# Supplementary material for: Population pharmacokinetics of intravenous and oral panobinostat in patients with hematologic and solid tumors
Source: Eur J Clin Pharmacol. 2015 May 5;71(6):663–72. doi: 10.1007/s00228-015-1846-7 (PMC4430599; doi:10.1007/s00228-015-1846-7)
Supplement: Supplementary file 8 — (DOC 40 kb) [file 228_2015_1846_MOESM8_ESM.doc]

Table S4: Variability of exposure metrics following a single dose of 20 mg for the two final models

| First Final Model |  |  |  |  |  |  |
| --- | --- | --- | --- | --- | --- | --- |
|  | min | Q1 | median | mean | Q3 | max |
| C_max_ (ng/mL) | 1.82 | 7.15 | 10.44 | 11.85 | 14.70 | 45.49 |
| C48h (ng/mL) | 0.04 | 0.28 | 0.51 | 0.72 | 0.97 | 4.02 |
| AUC0-48h (ng∙h/mL) | 16.60 | 61.42 | 92.12 | 111.58 | 145.37 | 383.39 |
| AUCinf (ng∙h/mL) | 18.07 | 77.77 | 121.44 | 165.70 | 212.71 | 1314.51 |
|  |  |  |  |  |  |  |
| Second Final Model |  |  |  |  |  |  |
|  | min | Q1 | median | mean | Q3 | max |
| C_max_ (ng/mL) | 2.32 | 7.76 | 9.81 | 11.02 | 13.49 | 37.49 |
| C48h (ng/mL) | 0.03 | 0.30 | 0.53 | 0.64 | 0.84 | 3.51 |
| AUC0-48h (ng∙h/mL) | 24.42 | 63.65 | 88.39 | 94.96 | 118.36 | 344.30 |
| AUCinf (ng∙h/mL) | 26.43 | 89.12 | 129.46 | 155.29 | 190.02 | 611.32 |

Note: Based on simulations of 300 patients with typical values of covariates (age 61 years, Caucasian; BSA = 1.9 m^2^ for first final model, weight = 76.4 kg for second final model)
